# Radiation Effects in Tungsten and Tungsten-Copper Alloys Treated with Compression Plasma Flows and Irradiated with He Ions

**DOI:** 10.3390/ma17184442

**Published:** 2024-09-10

**Authors:** Azamat Ryskulov, Vitaliy Shymanski, Igor Ivanov, Bauyrzhan Amanzhulov, Anastasia Dauhaliuk, Vladimir Uglov, Adilet Temir, Valiantsin Astashynski, Asset Sapar, Anton Kuzmitski, Yerulan Ungarbayev

**Affiliations:** 1 Institute of Nuclear Physics, Almaty 050032, Kazakhstan; igor.ivanov.inp@gmail.com (I.I.); amanzholovb96@gmail.com (B.A.);; 2 Department of Solid State Physics, Belarusian State University, 220030 Minsk, Belarus; shymanskiv@mail.ru (V.S.); dovgalyukna@gmail.com (A.D.); uglov@bsu.by (V.U.); 3 Engineering Profile Laboratory, L.N. Gumilyov Eurasian National University, Astana 010008, Kazakhstan; 4 Physical-Technical Faculty, L.N. Gumilyov Eurasian National University, Astana 010008, Kazakhstan; 5 A.V. Luikov Heat and Mass Transfer Institute of National Academy of Science of Belarus, 220072 Minsk, Belarus; ast@hmti.ac.by (V.A.);

**Keywords:** tungsten, copper, compression plasma flows, helium ions, ion irradiation, radiation effects, blistering

## Abstract

The paper presents the results of studying the structure and phase state of tungsten and tungsten-copper alloy after pulsed action of compression plasma flows and irradiation with helium ions. The compression plasma flows were used to modify the surface layer of tungsten, as well as to create an alloy based on tungsten and copper. Using scanning electron microscopy and X-ray structural analysis, the formation of radiation defects on the tungsten surface was detected in the form of local areas of exfoliation and destruction, which begin to form at helium ion irradiation doses of 2 × 10^17^ cm^−2^. It is shown that preliminary plasma treatment of the surface in the melting mode leads to the complete disappearance of surface radiation defects up to a dose of 2 × 10^17^ cm^−2^, which may be associated with the formation of a fine-crystalline grain structure, the intergranular boundaries of which serve as effective sinks for primary radiation defects.

## 1. Introduction

Tungsten-copper alloys are potential materials in electrical contacts, arcing electrodes, electronic packing, shielding materials, and others. The W-Cu composite materials are characterized by high electrical and thermal conductivities and low thermal expansion coefficient. However, there are some difficulties in the preparation of such alloys with high density. Currently, the main method of preparation is powder technology based on mixing tungsten and copper powders [1,2,3,4,5,6]. The poor wettability and immiscibility between tungsten and copper phases result in weak particle bonding. Due to a large difference in the melting point of tungsten (3422 °C) and copper (1080 °C), the most typical method of W-Cu composite preparation is based on the incorporation of liquid copper into the tungsten frame.

The W-Cu system features a largely positive heat of formation (+14 kJ/mole [7]) and exhibits total immiscibility in both solid and liquid states [8,9]. The formation of a W-Cu composite with inter-dissolving of both components is practically impossible by the equilibrium methods. Non-equilibrium techniques can be considered as synthesis methods for W-Cu metastable alloys or amorphous structures.

Improving the reliability, lifetime, and efficiency of materials in nuclear energy systems is an important issue requiring a deep theoretical and experimental study. The irradiation of the first wall with neutrons or helium ions can induce many material defects such as vacancies, interstitial clusters, helium bubbles, and voids [10,11,12,13,14]. These defects lead to volume swelling and blistering, which result in embrittlement and instability of the reactor components and reduce the lifetime. The ability to mitigate radiation-induced defects and control helium bubble nucleation and growth is crucial to improving radiation tolerance. It is known that a multiphase system with many interfaces can resist radiation damage because grain boundaries and interfaces act as sinks to annihilate point defects providing additional radiation tolerance compared to single-phase bulk materials [15].

The development and construction of thermonuclear (fusion) reactors are essential to the world’s energy system. One of the main persistent problems in fusion reactor construction is the selection of materials for plasma-facing components [16]. The materials must have high sputtering resistance, high melting point, low deuterium/tritium retention, high thermal conductivity, and superior mechanical properties. Tungsten is considered the main promising candidate for this role [17]. For example, the ITER program has been conducting research on tungsten-based first-wall components. On the other hand, copper is a promising material for heat sinks due to its high thermal conductivity. The tungsten monoblocks in the first wall and copper sinks must connect with the interlayer providing high thermal conductivity and low mechanical stress. Therefore, tungsten-copper alloys can be an effective solution to the interface problem [18].

In this paper, creating alloys based on tungsten and copper using compression plasma flows is proposed. This type of surface treatment is pulsed and allows transferring a sufficiently high energy density to the surface layer, which is necessary for its melting [19,20,21,22]. A feature of this type of treatment is the high cooling rates of the formed melt, which ensure that the processes related to the formation of the structure and phases during crystallization are non-equilibrium. Due to the high cooling rates, it is possible to achieve the condition of partial mixing of tungsten and copper in the liquid state, forming an alloy of a composite structure. In this work, it is proposed to use the effect of compression plasma flows in order to modify the structural state of the near-surface tungsten layer. Furthermore, in the case of the preliminary application of a copper coating to the surface of tungsten samples, exposure to compression plasma flows makes it possible to create an alloy based on tungsten and copper by means of their liquid-phase mixing.

Since tungsten alloys are considered materials for the first wall of thermonuclear reactors, it is vital to study their radiation resistance under conditions of long-term irradiation with helium ions, which are products of fusion reactions. In this regard, the main goal of this work is to establish the features of structural and phase changes in tungsten and tungsten-copper alloy caused by the impact of compression plasma flows and the effect of helium irradiation on the radiation resistance. Pure tungsten itself is considered a plasma-facing material. Tungsten-copper alloys are considered promising materials in heat sink elements in thermonuclear reactors [4,23]. The heat sink elements are not in contact with plasma and are not exposed to the He ions irradiation. However, many neutrons with large penetration depth are produced during the nuclear reactions. Therefore, the radiation effects can be found at a large depth under the surface. In this regard, the radiation effects in the tungsten-copper alloys were simulated with He ion implantation.

## 2. Materials and Methods

The experimental samples were made as plates of polycrystalline commercial pure tungsten with dimensions of 10 × 10 mm^2^ and a thickness of 2 mm. A thin copper coating with a thickness of 2 μm was deposited on the surface of the samples using the arc-vacuum technique.

The tungsten plates coated by Cu films were treated with compression plasma flows (CPF) in a magnetoplasma compressor of compact geometry. The compression plasma flow is obtained by a special device described in [24,25]. The plasma was generated due to discharge in the interelectrode space at a low-pressure atmosphere. The discharge was triggered between the central conical cathode and radially placed anodes. The discharge forms the plasma stream accelerated by Ampere force propagating at a high speed (50–70 km/s). The plasma stream is compressed by its magnetic field. A compression plasma flow of 6–12 cm in length with a diameter of 1 cm in the maximum compression zone was formed in the output of the discharge device. The tungsten samples were placed behind the compression region. The compression flow is stable during the time of the discharge, which is estimated as 100 µs; after that, the plasma flow starts to diverge.

The experiments were conducted in a “residual gas” mode in which a pre-evacuated vacuum chamber was filled with nitrogen gas up to a pressure equal to 400 Pa. The samples were placed vertically at the distance (*L*) from the electrode edge, which changed from 12 to 8 cm. It changed the plasma flow energy at the constant voltage (4.0 kV) between the electrodes. The diameter of the plasma stream in the target location is about 2–3 cm. For the material’s structure and phase composition modification by high-energy flow, the absorbed energy density (*Q*) transferred into heat is believed to be the main part of the total energy of the flow. Therefore, the absorbed energy density transferred from the CPF to the tungsten samples during the impact was measured by a calorimetric method. The dependence of the absorbed energy density on the distance *L* can be found in the work [26]. The highest absorbed energy density reached 70 J/cm^2^ at *L* = 6 cm and dropped to 35 J/cm^2^ at *L* = 10 cm. The samples were treated with three CPF pulses with a time between each pulse of 20–30 s.

The elemental composition as well as phase composition and surface morphology of the W-Cu alloys were analyzed after the CPF impact. The elemental composition of the treated samples was determined by energy-dispersive X-ray microanalysis (EDX) with the Oxford Max^N^ analyzer (Oxford Instruments, Oxford, UK). The phase composition of the modified layer in the tungsten was investigated by the X-ray diffraction (XRD) method with Ultima IV RIGAKU diffractometer (Rigaku, Tokyo, Japan) in the Bragg-Brentano geometry with parallel beams in Cu Kα radiation (λ = 0.154178 nm). The error in the lattice parameters determination with the XRD method did not exceed 0.1%. The surface morphology was analyzed by scanning electron microscopy (SEM) with the LEO 1455 VP microscope (Carl Zeiss, Jena, Germany) at an accelerating voltage of 20 kV.

The samples were irradiated at a DC-60 heavy ion accelerator at the Astana branch of the Institute of Nuclear Physics (Astana, Kazakhstan) [27]. The targets were irradiated in an experimental chamber for irradiating physical targets with low-energy ion beams, equipped with all the necessary equipment. During irradiation, the studied samples were placed on a water-cooled target holder to eliminate the influence of thermal heating due to the accumulation of the irradiation dose. The target irradiation was carried out under high vacuum conditions maintained at a level of (1–5) × 10^−5^ Torr. Irradiation was carried out with He^2+^ ions with an energy of 40 keV at fluences of 1 × 10^17^ cm^−2^, 2 × 10^17^ cm^−2^, and 3 × 10^17^ cm^−2^. This type of ion emerges in reactors as a result of the interaction of neutrons with atoms of matter, followed by alpha decay, which leads to the formation of gas bubbles, areas of increased internal stresses, accumulation of helium in the material, and, as a result, to its swelling. The parameters of the helium ions irradiation are presented in Table 1.

## 3. Results and Discussion

### 3.1. Compression Plasma Flows

Compression plasma flows are generated by quasi-stationary plasma accelerators, where plasma is accelerated due to its interaction with its magnetic field. A characteristic feature of used plasma flows is a unique combination of high energy density and a sufficiently long plasma pulse duration. In terms of using pulsed plasma action on the surface of materials, the most important parameter is the density of thermal energy, which is absorbed by the near-surface layer. It is a part of the total energy of the plasma flow that contributes to structural transformations in the material. For material modification and multicomponent alloy production, the plasma parameters corresponding to high absorbed energy density are more promising. The absorbed energy density is sufficiently high for the melting of a thick (up to tens of micrometers) surface layer, which, combined with a long pulse duration, initializes the mixing of the liquid state. When acting on a coating/substrate system, plasma flow melts and mixes both the coating metal and substrate parts together.

Considering the values of the absorbed energy and pulse duration, the temperature distributions in the surface top layer of the tungsten were calculated [26]. Melting of the tungsten surface begins at the absorbed energy density of 55 J/cm^2^ and higher. According to the results presented in [19,26], the depth of the molten tungsten layer after plasma treatment reaches 5 μm with an absorbed energy density of 55 J/cm^2^. The results presented in these works allow us to conclude that the effect of CPF on the surface of tungsten and tungsten coated with copper leads to the heating of the surface layer to a temperature of 3500–3700 K, which is sufficient for melting the surface layer. On the other hand, copper will intensively evaporate after such a strong plasma impact. Therefore, the experiments with lower energy density were carried out.

**Figure 1:**
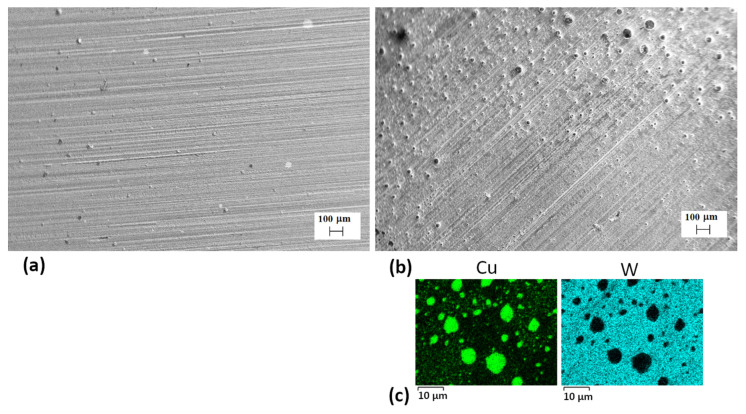
SEM-images of the surface of W-Cu samples: (**a**) before and (**b**) after CPF at *Q* = 35 J/cm^2^, (**c**) EDX maps of W-Cu after CPF. In Figure 1b, spherical particles on the surface are Cu particles.

The result of plasma acting on the surface can be analyzed using the SEM method. The SEM image of the surface of the tungsten with a copper coating before the plasma treatment is presented in Figure 1a. The presence of many parallel lines is a result of surface polishing before the coating deposition. The copper surface morphology repeats the roughness of the plate. After the plasma impact at the absorbed energy density of 35 J/cm^2^, many spherical particles are observed (Figure 1b). According to the EDX analysis (surface mapping), each particle contains pure copper. The sizes of the copper particles belong to the range 1–3 μm, some of them reach the size of 10 μm. The absorbed energy density of 35 J/cm^2^ exceeds the melting threshold for copper but is not sufficient for tungsten melting. Thus, the molten copper produces spherical particles due to the surface tension as well as bad wetting of tungsten by copper, which becomes solid after crystallization. Figure 1c shows the presence of copper on the tungsten surface between the particles. It can be a result of condensation of copper after the end of the plasma flow impact. Indeed, the high energy of the plasma flow results in evaporation and ablation of the material facing the stream. The products of the evaporation cannot move far from the target because of the compressive action of the oncoming plasma stream and accumulate in the shock-compressed layer near the surface. After 100 μs of pulse duration, the plasma flow diverges and the elements from the shock-compressed layer re-condensate on the surface.

**Figure 2:**
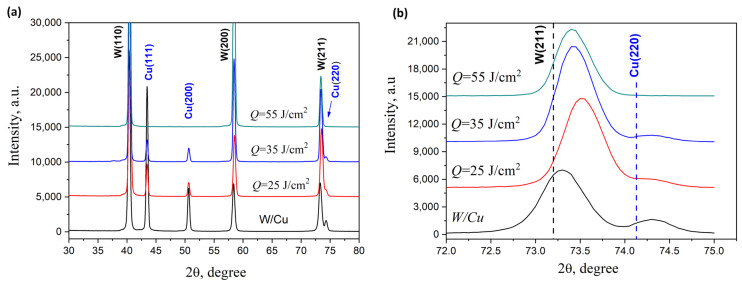
(**a**) XRD patterns and (**b**) magnified XRD peaks of the W-Cu samples after CPF at different absorbed energy densities.

Figure 2 shows the XRD results and the phase composition of the W/Cu system before the plasma treatment. Two separate phases bcc W and fcc Cu with polycrystalline structure were found. The lattice parameters of the phases agree with the standard values: *a*_W_ = 0.3163 nm (*a*_0_ = 0.31648 nm (JCPDS no. 00-004-0806)) for tungsten and *a*_Cu_ = 0.3611 nm (*a*_0_ = 0.3615 nm (JCPDS no. 00-004-0836)) for copper. The double-phase system in the surface layer of the W-Cu samples is preserved after the compression plasma flow impact. However, the intensities of the copper diffraction lines decrease with the absorbed energy density growth from 35 to 55 J/cm^2^. It is a result of an increase in the evaporated fraction of copper due to the high temperature on the surface. There are no diffraction peaks of copper in the W-Cu samples after plasma treatment with the absorbed energy density of 55 J/cm^2^. The positions of the copper peaks in the XRD patterns do not change with a change in the energy density parameter of the plasma flows. This means that the lattice parameter of the copper particles is stable and there is no dissolution of tungsten atoms in the crystal lattice of copper. Nevertheless, the lattice parameter of tungsten slightly decreases to 0.3157 nm after the plasma treatment, which can be seen in the magnified part of the XRD patterns (Figure 2b). The change in the tungsten lattice parameter could be due to the excess of point defects, such as vacancies, after crystallization. Indeed, a crucial result of the pulsed treatment of the metal surfaces is a quenching effect due to the high solidification rate. When the heat transfer from the plasma stream stops, cooling of the melted layers occurs. Because of a strong correlation between the thickness of the non-molten metal part and the molten surface layer, the cooling rate can reach 10^6^–10^7^ K/s [20,21,22]. The quenching effect produces an excess concentration of vacancies, which can decrease the lattice parameters of the crystal structure in the modified metals. However, the results of the effect of plasma with the same energies on pure tungsten, without any other metal as a coating, showed constant values of its lattice parameters [26]. Thus, it can be assumed that the presence of copper atoms in the crystal lattice of tungsten results in solid solution W(Cu) formation. The atomic radius of copper (128 pm) is less than that of tungsten (141 pm), and the formation of W(Cu) solid solution can be associated with its lattice parameter decrease. The shift of the diffraction peaks of tungsten after the compression plasma flow impact depends on the copper content in the crystal lattice. Therefore, for the heat load with the absorbed energy density of 25 J/cm^2^, the shift is higher because of the strong evaporation of copper at higher energies.

Despite using nitrogen as a plasma-forming gas, nitride phases based on tungsten or copper were not found.

It should be noted that with an increase in the absorbed energy density, the integral intensity of the diffraction lines of copper decreases, which indicates a decrease in its volume content in the analyzed layer, and at the maximum absorbed energy density, Cu diffraction lines were not detected. Thus, according to the results of the elemental analysis carried out by X-ray spectral microanalysis, the copper content in the near-surface layer of the sample formed at the minimum absorbed energy density (25 J/cm^2^) is 62.5 atomic percent (at.%) and at the maximum absorbed energy density (55 J/cm^2^) it decreases to 1.3 at.%, which may indicate partial erosion of the surface with subsequent removal of copper due to evaporation, ablation, or hydrodynamic spreading.

### 3.2. Ion Irradiation of Tungsten and the Tungsten-Copper Alloy

Tungsten samples processed by CPF were irradiated with helium ions with an energy of 40 keV. The samples with tungsten-copper alloy produced at the CPF influencing the absorbed energy density 55 J/cm^2^ were chosen for irradiation with helium ions. According to the results of ion path modeling obtained in the Stopping and Range of Ions in Matter (SRIM-2013) [28] program, the depth that the ion range reaches in tungsten does not exceed 400 nm, significantly less than the thickness of the layer modified by plasma exposure. The depth of the layer modified by CPF impact is estimated as 5 μm. It is in this layer that the formation of a W(Cu) solid solution is expected, which corresponds to the depth of the molten layer after plasma exposure. Thus, it can be assumed that the detected radiation effects in the surface layer of tungsten are affected by structural changes caused by plasma treatment.

**Figure 3:**
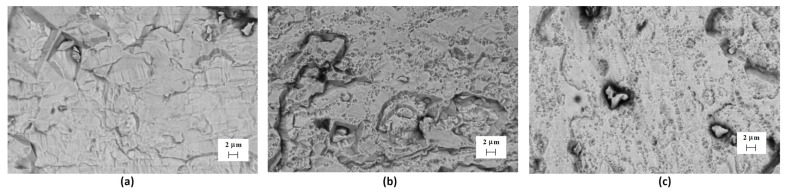
SEM images of the tungsten surface after He ion irradiation: (**a**) D_1_ = 10^17^ cm^−2^, (**b**) D_2_ = 2 × 10^17^ cm^−2^, and (**c**) D_3_ = 3 × 10^17^ cm^−2^.

**Figure 4:**
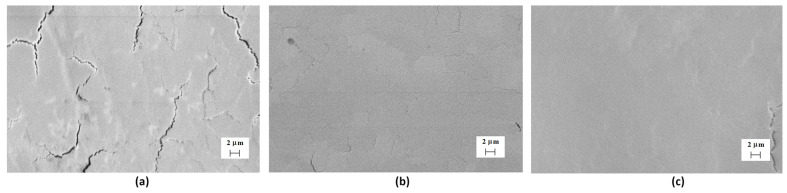
SEM images of the tungsten surface after plasma treatment and He ion irradiation: (**a**) D_1_ = 10^17^ cm^−2^, (**b**) D_2_ = 2 × 10^17^ cm^−2^, and (**c**) D_3_ = 3 × 10^17^ cm^−2^.

Irradiation of the tungsten sample surface with helium ions primarily results in a change in the surface morphology. Figure 3 shows the SEM images of the tungsten surface in its original state, without plasma treatment, but irradiated with helium ions. The results indicate a change in the surface morphology of the original state, starting with a dose of 2 × 10^17^ cm^−2^. The images show many small areas up to 1 μm in size, which appeared as a result of ion sputtering of the surface. Irradiation of the tungsten surface pre-treated with compression plasma flows with helium ions with similar energies and doses does not result in surface sputtering, and the surface morphology remains unchanged over the entire range of doses used (Figure 4). The only difference in the morphology of the treated tungsten is the presence of microcracks formed due to high-temperature heating of the surface and rapid cooling, which lead to the formation of a high level of thermoelastic stresses exceeding the tensile strength of the material in local areas. You can also notice a smoothing of the tungsten surface relief after plasma treatment, which is a consequence of liquid-phase processes on the molten surface.

**Figure 5:**
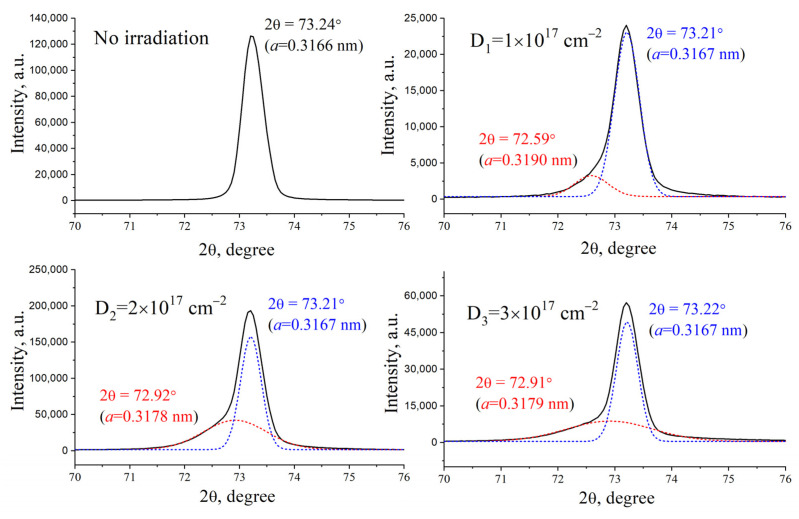
XRD profile of the (211) W line for the initial tungsten samples irradiated with He ions.

**Figure 6:**
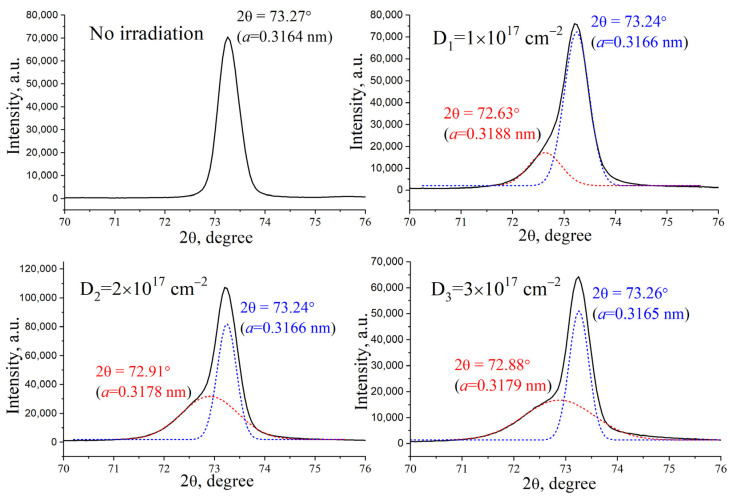
XRD profile of the (211) W line for the tungsten samples treated with CPF and irradiated with He ions.

Using X-ray structural analysis, a change in the tungsten crystal structure was established after ion irradiation (Figure 5 and Figure 6). Qualitatively, the phase composition of the tungsten samples did not change after exposure to compression plasma and ion irradiation; only tungsten diffraction lines were detected on the X-ray patterns. The diffraction line (211) was selected to analyze the change in the tungsten lattice parameter after treatment. The experimental data demonstrate a change in the symmetry of the peak (211) associated with the appearance of a low-angle shoulder. Indeed, the mathematical deconvolution of the experimentally obtained diffraction peak showed the presence of two different phases. The diffraction peak from the side of large diffraction angles, which has a high intensity, corresponds to the tungsten region that was not exposed to ion irradiation. Since the depth of analysis using X-ray diffraction is about 1 μm, the formation of this peak in the diffraction pattern is due to reflection from the layers below the implanted layer. The analysis of the tungsten lattice parameter, performed along the diffraction line from the side of large diffraction angles, showed values of 0.3166–0.3167 nm, which do not change at all the irradiation doses used. The appearance of the small-angle diffraction peak is due to X-ray reflection from the implanted region, in which a slight distortion of the crystal lattice occurs. For the original tungsten, the lattice parameter in the implanted area decreases from 0.3190 to 0.3179 nm with an increase in the dose of helium ions from 1 × 10^17^ cm^−2^ to 3 × 10^17^ cm^−2^.

It should be noted that the detected values of the lattice parameter are higher compared to the lattice parameters in the non-implanted area. The main primary result of ion irradiation is the accumulation of helium ions in the interstitials, and the formation of their complexes and clusters with an increase in the implanted dose, which leads to an increase in the lattice parameter due to its stretching. It can be seen that the system irradiated with helium ions at a minimum dose of 1 × 10^17^ cm^−2^ has the maximum value of the lattice parameter, which indicates the presence of a high level of residual stresses in the structure. With a further increase in the dose of implanted helium ions, there is an increase in the distortions of the crystal lattice and, as a consequence, an increase in residual stresses. Relaxation of these stresses causes mechanical peeling of microscopic areas, leading to a change in the surface morphology detected by the SEM method. The lattice parameter of tungsten at the highest dose slightly decreases to 0.3179 nm.

Similar changes in the tungsten crystal lattice parameters are found in the near-surface layer after preliminary plasma treatment. With an increase in the dose of implanted helium ions from 1 × 10^17^ cm^−2^ to 3 × 10^17^ cm^−2^, the tungsten lattice parameter changes from 0.3188 to 0.3179 nm. However, no changes in the surface morphology were detected. It can be assumed that there is no relaxation of internal stresses, and no peeling is observed. A feature of materials, in particular tungsten, treated with pulsed high-energy CPF, is the formation of a near-surface layer as a result of high-speed quenching from a molten state. In this case, cooling rates of 10^6^–10^7^ K/s are achieved, contributing to the growth of a fine-grained structure with an increased proportion of intergrain boundaries. It can be assumed that the high proportion of such grain boundaries in tungsten samples after preliminary plasma treatment allows the level of residual stresses in the crystal lattice to be removed due to the flow of interstitial atoms to the boundaries themselves. The saturation effect in the lattice parameter change in the tungsten is observed. This effect can be explained by the critical internal mechanical stress, which facilitates the destruction of the surface by radiation effects. The incorporation of the helium ions in the tungsten crystal lattice changes the lattice parameter and increases the stress. After the critical level, the stress can lead to a local destruction of the surface with a small effect of flacking.

**Figure 7:**
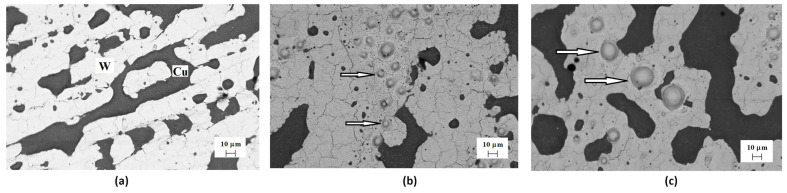
SEM images of the copper-tungsten surface after CPF treatment and He ion irradiation: (**a**) D_1_ = 10^17^ cm^−2^, (**b**) D_2_ = 2 × 10^17^ cm^−2^, and (**c**) D_3_ = 3 × 10^17^ cm^−2^. Dark areas—Cu, light areas—W. The blisters on the surface are denoted by arrows.

As described earlier, compression plasma flows were used to create an alloy based on tungsten and copper. The results of irradiating samples of such an alloy with helium ions are shown in Figure 7. In the image of the surface, dark and light areas can be observed, which correspond to copper and tungsten, respectively. The appearance of such delimited areas is a consequence of the immiscibility of tungsten and copper in the liquid state. The irradiation of tungsten-copper alloy with helium ions at doses of (2–3) × 10^17^ cm^−2^ leads to fairly large bubbles (blisters), localized mainly in the tungsten region. Such blisters are not detected in the copper areas. The average size of blisters formed at an irradiation dose of 2 × 10^17^ cm^−2^ is 5–10 μm, and increases to 15 μm with an increase in the dose of implanted ions to 3 × 10^17^ cm^−2^.

**Figure 8:**
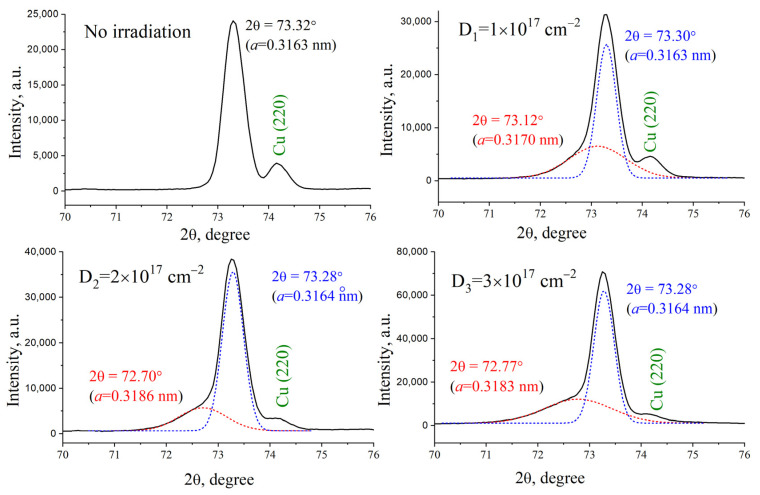
XRD profile of the (211) W line for the copper-tungsten samples treated with CPF and irradiated with He ions.

The analysis of the structural state of W-Cu (Figure 8) also showed the presence of a distorted tungsten lattice in the near-surface region, and, unlike unalloyed tungsten samples, the tungsten lattice parameter increases from 0.3170 to 0.3186 nm with an increase in the dose from 1 × 10^17^ cm^−2^ to 2 × 10^17^ cm^−2^. An increase in the lattice parameter indicates an increase in residual stresses in the tungsten crystal structure, which does not have time to relax. The accumulation of helium ions in local areas leads to the formation of blisters, but the increased elastic parameters of the modified tungsten-copper alloy layer do not allow their mechanical destruction. The reason for such a change in the elastic parameters of the tungsten regions in the alloy can be the following. The insignificant dissolution of copper in the tungsten lattice, as well as the presence of interphase boundaries between individual phases of tungsten and copper, together lead to an increase in compressive internal stresses that prevent the destruction of the surface after ion irradiation.

## 4. Conclusions

Overall, the work demonstrated the fundamental opportunity of creating alloys based on tungsten and copper by successively applying copper coating to the tungsten surface and using pulsed action of compression plasma flows with a pulse duration of 100 μs, with an absorbed energy density of 35–55 J/cm^2^. The results demonstrate the formation of a composite structure based on individual phases of tungsten and copper, and due to the high-speed crystallization of the molten surface layer, a solid solution of W(Cu) with a distorted crystal lattice is formed. Analysis of radiation effects during irradiation of tungsten and tungsten-copper alloy samples with helium ions with an energy of 40 keV and doses of (1–3) × 10^17^ cm^−2^ showed that tungsten samples pre-treated with CPF in the melting and mixing mode of the near-surface layer have increased radiation resistance. In the near-surface region, corresponding to the helium ion range, there is a distortion of the tungsten phase crystal lattice, which causes mechanical destruction of the surface. Preliminary modification of the tungsten surface by CPF creates a finely dispersed structure of the surface layer, where the increased number of intergranular boundaries mitigates the effect of surface destruction due to the migration of radiation defects to the boundaries. The formation of an alloy based on tungsten and copper using compression plasma flows leads to a more significant change in the surface morphology after irradiation with helium ions with similar energies and doses. The formation of blisters is observed on the surface of the alloys, which are absent in the case of irradiation of an undoped tungsten sample.

## Figures and Tables

**Table 1:** Parameters of the ion irradiation of W, W-Cu targets.

Ions	Energy, keV	Temperature of Irradiation, °C	Flux, cm^−2^·s^−1^	Irradiation Time	Fluence, cm^−2^
He^2+^	40	25	9.11 × 10^12^	3 h 3 min	1 × 10^17^
He^2+^	40	25	1.10 × 10^13^	5 h 2 min	2 × 10^17^
He^2+^	40	25	1.05 × 10^13^	7 h 57 min	3 × 10^17^

## Data Availability

The authors declare that the data supporting this study are available from the corresponding author upon request.
